# Late detachment of ascending aortic graft mimicking pseudoaneurysm and dissection: A case report

**DOI:** 10.1016/j.radcr.2023.07.067

**Published:** 2023-08-17

**Authors:** Renato Fabrizio, Laura Mascitti, Sara Boemi, Sara Pasi, Matteo Giavarini, Martina Conca

**Affiliations:** Postgraduation School in Radiodiagnostics, Università degli Studi di Milano, Via Festa del Perdono 7, 20122, Milan, Italy

**Keywords:** Ascending aorta aneurysm, Aorta graft, Pseudoaneurysm, Aortic dissection, Cardiovascular imaging

## Abstract

Ascending aorta aneurysm is a pathological dilatation of the aortic wall which needs in most cases surgical treatment.

Complications after surgery are usually rare events and include infections, bleeding, and pseudoaneurysm.

We present a report of a late complication after ascending aorta aneurysm repair consisting of a prosthetic detachment and dislocation miming a pseudoaneurysm of the aortic root associated with an aortic dissection.

This case was radiologically challenging due to the atypical CT aspect and therefore it required a deep radiological, clinical, and surgical analysis with a multidisciplinary approach.

## Introduction

Ascending aorta aneurysm is defined as a pathological dilatation of the aortic wall due to intrinsic weakness that can involve the tubular part of the ascending aorta (above the sinotubular junction), the aortic root (below the sinotubular junction) or both.

The most common risk factors are age-related degeneration, genetic elastopathies (such as Marfan Syndrome), and bicuspid aortic valve (BAV) [1].

Diagnosis is commonly based on Transthoracic echocardiography (TTE) and on enhanced CT scan performed with an electrocardiogram (ECG)-gated acquisition protocols, which plays a central role in the diagnostic flowchart because it allows accurate aortic evaluation and measurements [2,3].

Treatment could be either medical or surgical. Indications for surgery are based mainly on aortic diameter and the patient's clinical conditions. Surgery should be considered when the diameter is ≥ 55 mm for any patient when the diameter is ≥ 50 mm in the case of Marfan Syndrome (and should be even considered when the diameter is ≥ 45 mm in the presence of other risk factors) and in patients with BAV and other risk factors (including hypertension and aortic regurgitation) [3].

Various surgical techniques may be used to reconstruct the aorta depending on which part of the ascending aorta the aneurysm involves. If the dilatation is proximally limited to the sinotubular junction and distally to the aortic arch, a valve-sparing procedure such as the T. David–V technique could be chosen. In case of aortic valve involvement, the wheat procedure can be an option, in which both an ascending aortic graft and an aortic valve prosthesis are separately implanted. If the aneurysm extends below the sinotubular junction, the surgical repair is guided by the extent of involvement of the aortic annulus and the aortic valve with a composite synthetic ascending aorta and aortic valve graft placed together, as in the Bentall-De Bono procedure [4,5].

Patients with treated ascending aorta aneurysms should be followed up with serial imaging studies for surveillance based on TTE and enhanced CT. The exact timeline of surveillance depends on the aneurysm size, evidence of growth, and associated clinical conditions [3,6].

The following case reports an example of a treated ascending aorta aneurysm with a long-term postprocedural complication.

## Case report

**Fig. 1:**
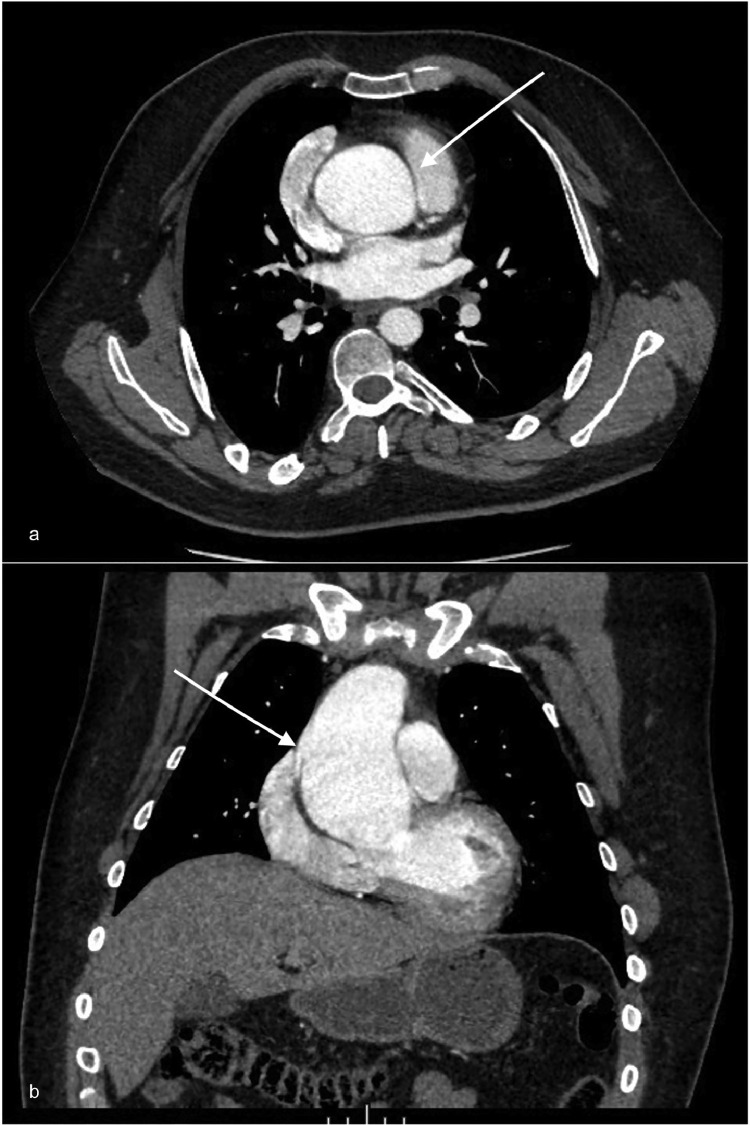
Original ECG-gated enhanced CT scan performed in 2012 showing on both axial (A) and coronal (B) plane an aneurysmatic dilatation involving the tubular part of the ascending aorta with sparing of the sinotubular junction and of the aortic root (white arrows). The maximum diameter reported was 57 mm sampled at the middle third of the ascending aorta using an MPR (multi plane reformation) tool.

A 54-year-old man received a diagnosis of ascending aorta aneurism limited to the tubular part by the end of 2012, as shown in Fig. 1. He was treated in February 2013 with an ascending aortic graft and an aortic valve prosthesis implant (wheat procedure).

No complications were evident in the postprocedural imaging and he continued regular imaging follow-ups with yearly enhanced CT scans and TTE (6 months apart), which were always unremarkable.

The patient has always been completely asymptomatic, with no dyspnea, chest pain, or palpitation reported.

The last TTE has highlighted severe aortic root dilatation. The ascending aortic graft was nonevaluable, whereas the aortic valve prosthesis was normo-positioned and normo-functional.

**Fig. 2:**
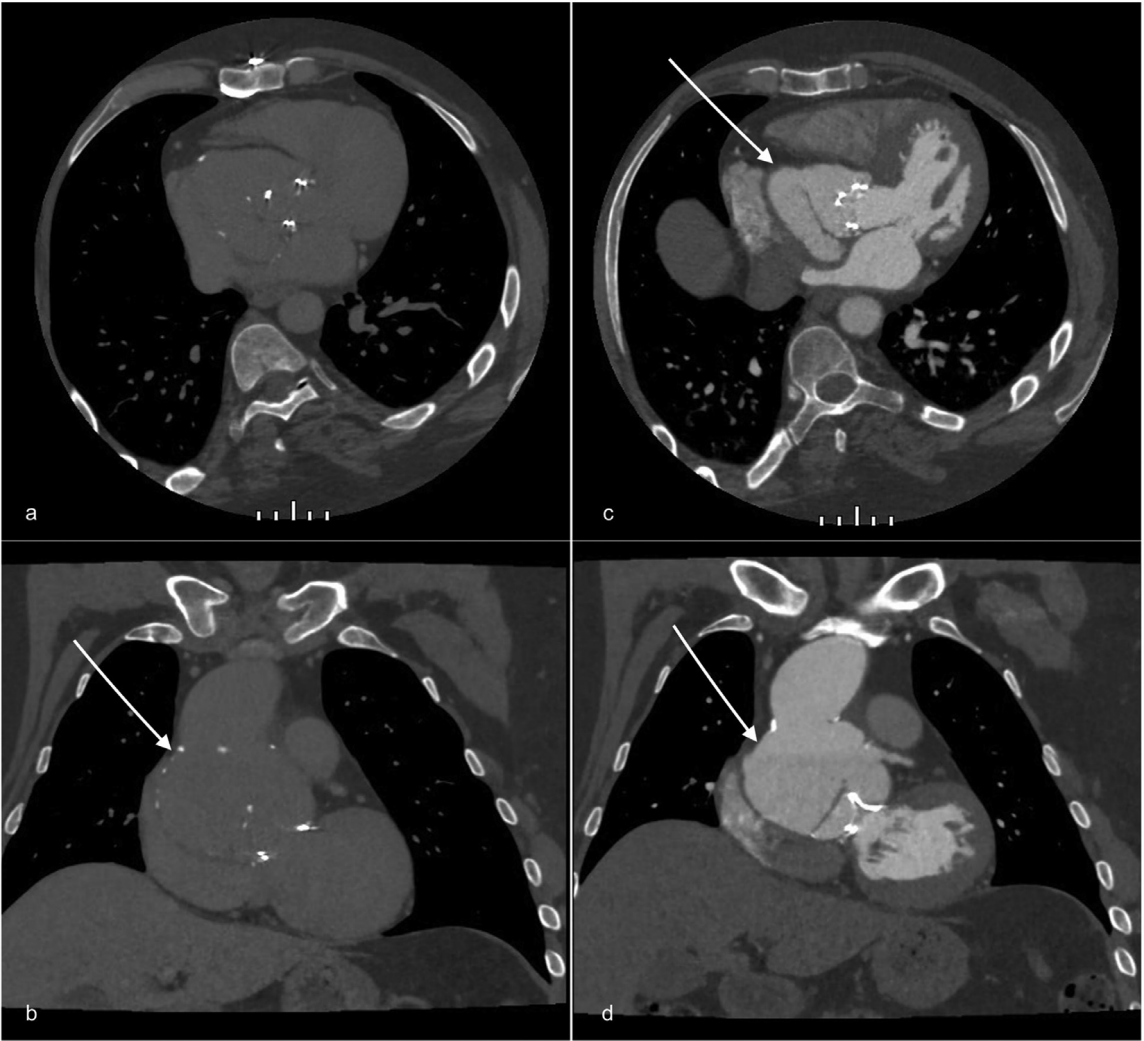
Preoperatory ECG-gated CT scan on axial (A, C) and coronal (B, F) planes before (A, B) and after contrast agent injection (C, D) showing the severe dilatation of the aortic root extending to the aortic bulb and the ascending aorta (white arrows). The graft is not visible; just the aortic valve prosthesis is recognizable. These findings resembled a pseudoaneurysm.

**Fig. 3:**
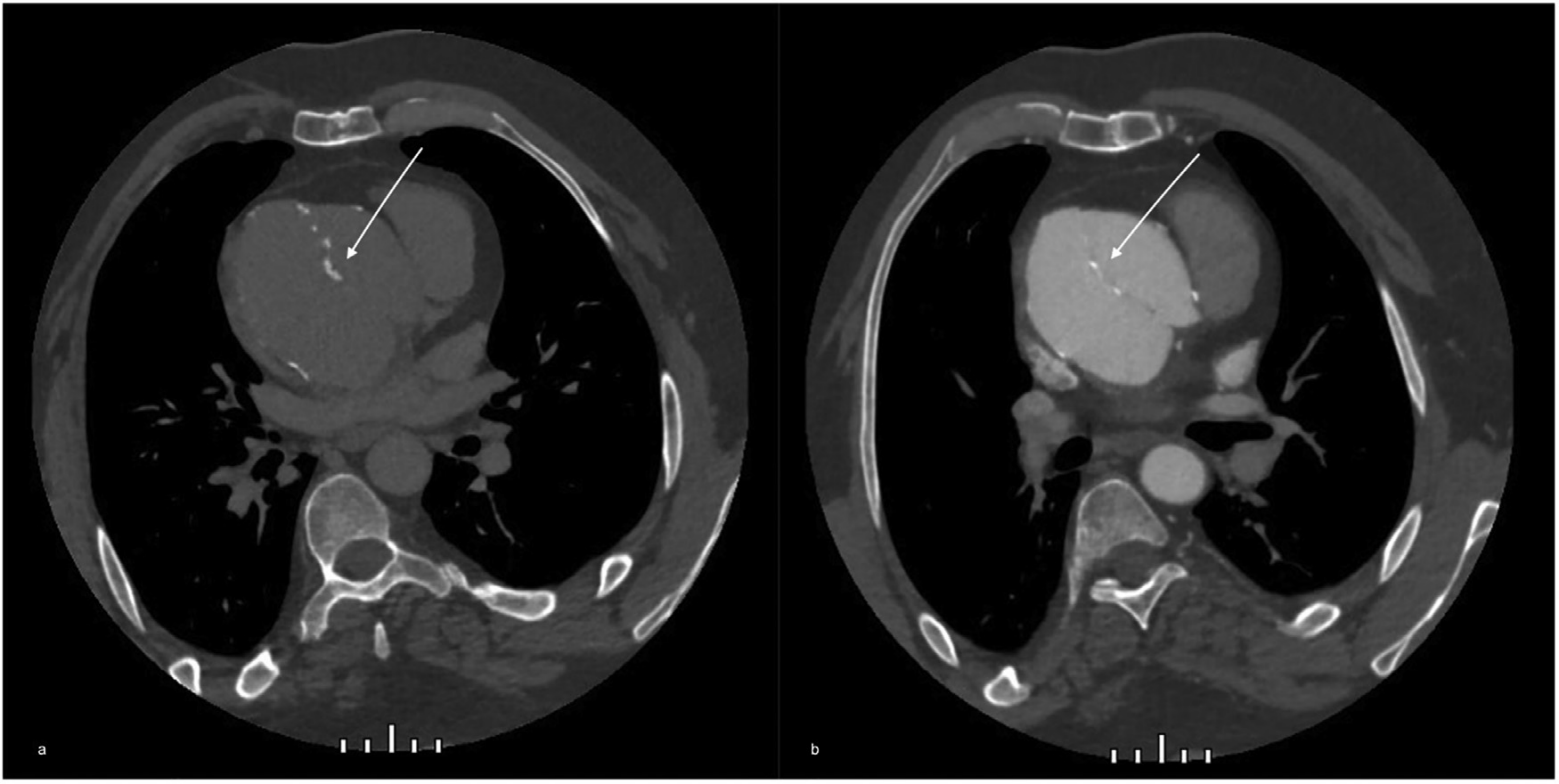
Unenhanced (A) and enhanced (B) axial image of the same preoperatory CT shown in Figure 2 with a focus on the “flap-like” aspect (white arrows). It can be seen a small septum with calcific spots that seemed to divide the aortic lumen into 2 cavities.

**Fig. 4:**
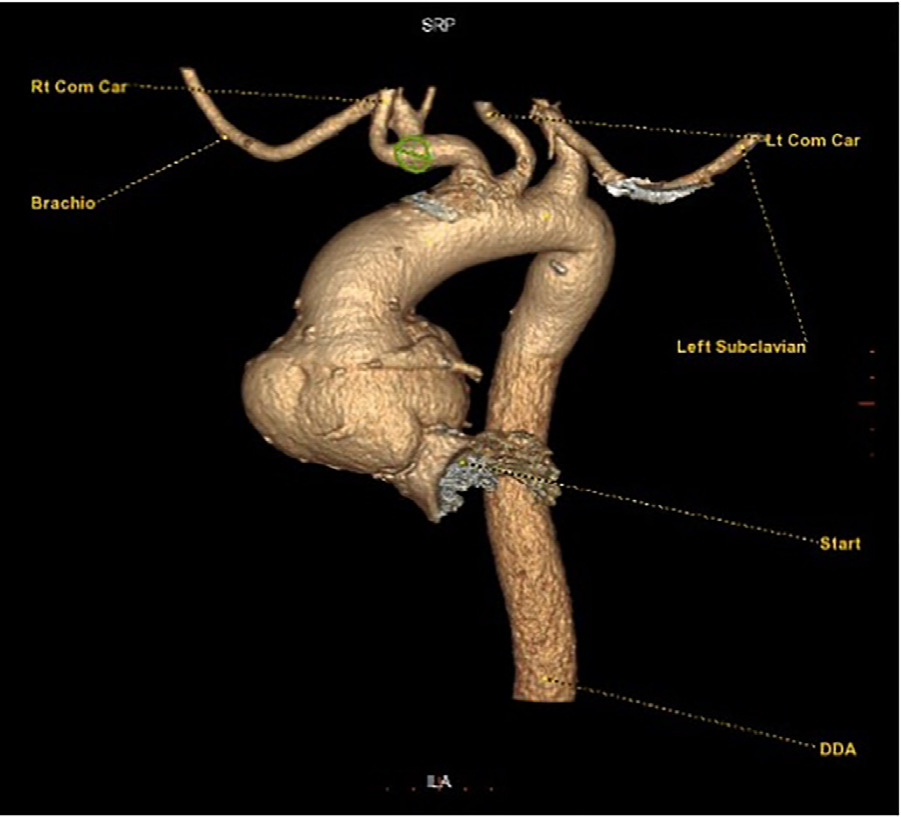
A 3D (three dimension) VR (volume rendering) image created on the preoperatory CT scan showing the localization of the aneurysm at the aortic root level.

Therefore, a new enhanced CT scan was performed. It confirmed the severe aortic root dilatation, showing also a diffuse ascending aorta and an aortic bulb dilatation associated with an intimal flap originating from the left coronary sinus to the origin of the anonymous trunk (Figs. 2–4).

The final diagnosis was a periprosthetic pseudoaneurysm associated with a possible initial proximal and distal perianastomotic dissection regarding the bulb and the distal part of the native ascending aorta attached to the aortic arch.

The patient was shortly evaluated by the cardiothoracic surgery team and discussed in a multidisciplinary meeting involving cardiac surgeons, cardiologists, radiologists, and anesthetists, who decided for urgent open re-operation.

The patient was conducted to the operating room for a transverse aortoromy of the graft under general anesthesia and extracorporeal circulation.

During the procedure, it was discovered that the graft was completely detached both proximally and distally from the aortic bulb and the distal ascending aorta respectively, and that the aortic root dilatation reported was a proper aneurismatic dilatation caused by the graft dislocation.

The patient was treated performing the Bental-De Bono procedure with a graft replacement of the entire ascending aorta, including the aortic root, re-implantation of the coronary arteries into the graft, and an aortic valve prosthesis replacement.

**Fig. 5:**
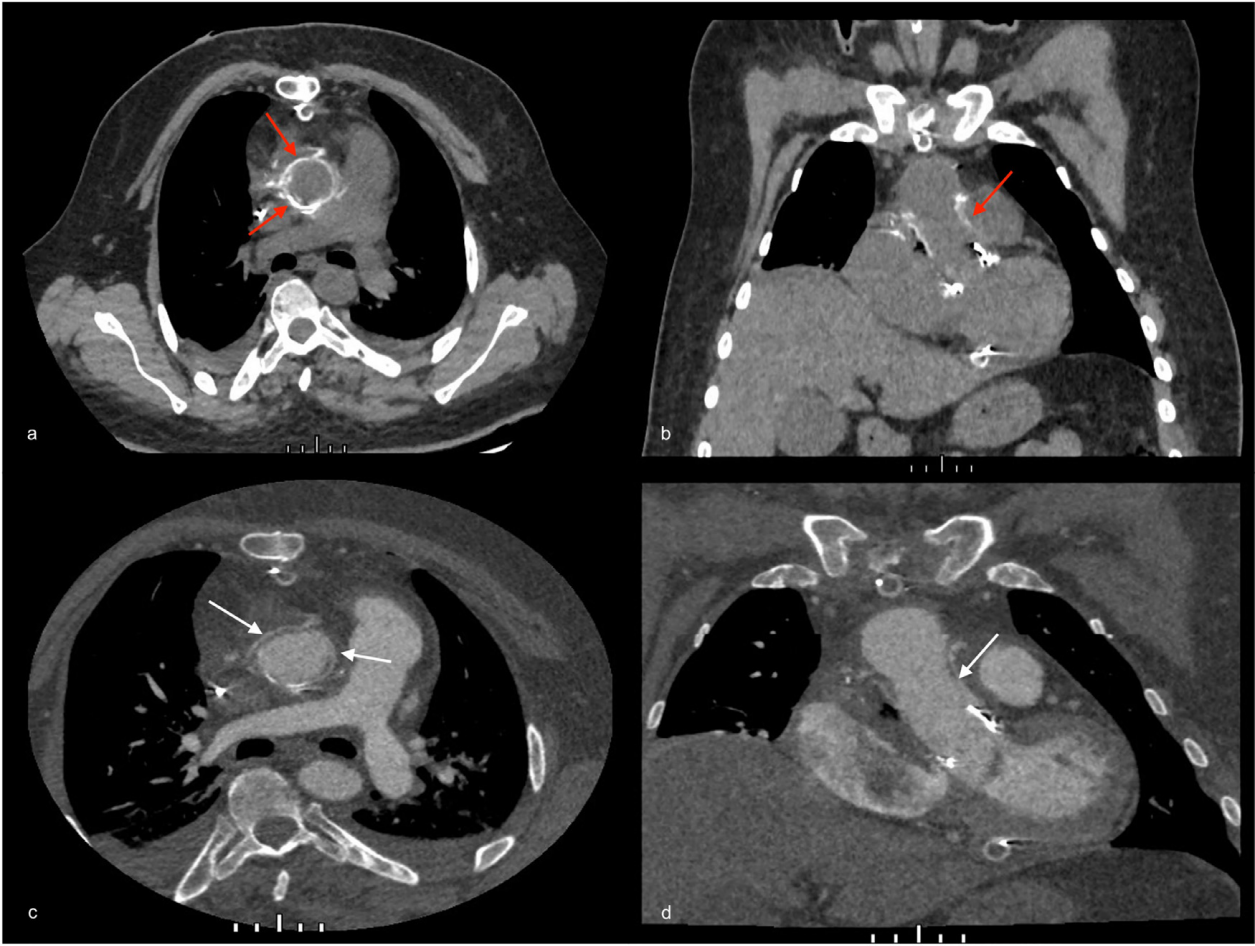
Postoperatory CT scan performed 4 days after surgical treatment. The new ascending aortic graft appears strongly hyperdense on unenhanced images (A, axial; B, coronal; red arrows) and becomes hypodense on enhanced arterial phase images (C, axial; D, coronal; white arrows). No local complications are evident.

Another enhanced CT scan was performed some days after surgery and no complications have been highlighted (Fig. 5).

The patient has been therefore discharged in good clinical condition with antihypertensive therapy, a cardiorespiratory rehabilitation program, and clinical and imaging checks 3 months apart.

## Discussion

We reported this case because it shows an atypical radiological aspect of a rare late postoperative complication of ascending aorta aneurysm repair.

Some of the most frequent complications of ascending aorta repairs, particularly via open surgery, are mediastinal infections and periprosthetic hemorrhages and hematomas, which usually compare some weeks after surgery [4,7].

Among late complications of ascending aorta aneurysm repair, pseudoaneurysm is the most frequent. Paranastomotic pseudoaneurysm is a rare but potentially fatal event that occurs in less than 0.5% of cases after cardiac surgery, usually associated with mediastinitis and graft infections and its typical location is the graft anastomosis site [4,7].

Other extremely rare late complications of ascending aorta aneurysm repair are dissection and evolutive aortic aneurysms developing at distant locations from the graft attachment [8].

The interesting thing about this case is that the aneurysmatic dilatation has involved the proximal part of the graft due to a complete detachment of the graft itself involving also the aortic root area (Figs. 2 and 4).

In fact, Chu et al. confirm in their work that a progressive aortic root aneurysm or dissection, although rarely, may develop, in patients with a history of aortic root repair. The wheat procedure originally performed on our patient spared the aortic root, so an aneurysm was not probable and therefore the diagnostic hypothesis shifted to pseudoaneurysm, which is much more frequent [8].

Moreover, the dislocated prosthesis, once removed, was found very damaged and degenerated and this deeply changed its aspect on CT images, making it just barely hyperdense, with tiny spots on unenhanced CT and with no significant changes after contrast agent injection, resembling a dissection flap (Fig. 3).

In fact, most of the surgical materials used in cardiac surgery, such as felt strips, grafts, and pledgets, have a very high attenuation on unenhanced CT miming contrast material, but they are usually not visible on enhanced images [4,9].

This different aspect between unenhanced and enhanced CT can be seen by comparing images in Figs. 2 and 5.

## Conclusion

Patients with a history of repair of the ascending aorta performing a CT represent a strong challenge for the reporting radiologist.

In fact, this type of examination is not carried out routinely in all centers due to a lack of technology and adequate skills, and evaluation of complications after surgery, rare occurrences already in itself, can be extremely complex.

Deep knowledge of the most commonly used repair components and employed repair techniques is mandatory for radiologists in order to correctly interpret imaging findings.

In a case as complicated as the one shown, a multidisciplinary approach and discussion involving surgeons and other specialists is preferable: this may lead to a better clinical and diagnostic setting of the patient and consequently to the best therapeutic choice, customized to the type of problem highlighted.

## Patient consent

Written, informed consent was obtained from the patient for publication of this case.
